# Cognitive performance of premature infants: association between bronchopulmonary dysplasia and cognitive skills. Cross-sectional study: Desempenho cognitivo de prematuros: associação da displasia broncopulmonar com as habilidades cognitivas. Estudo transversal

**DOI:** 10.1590/1516-3180.2017.0010190317

**Published:** 2017-07-31

**Authors:** Rosane Reis de Mello, Ana Beatriz Rodrigues Reis, Kátia Silveira da Silva

**Affiliations:** I MD, PhD. Attending Physician, Department of Neonatology, Instituto Nacional de Saúde da Mulher, da Criança e do Adolescente Fernandes Figueira, Fiocruz, Rio de Janeiro (RJ), Brazil.; II MSc. Clinical Psychologist, Department of Neonatology, Instituto Nacional de Saúde da Mulher, da Criança e do Adolescente Fernandes Figueira, Fiocruz, Rio de Janeiro (RJ), Brazil.; III MD, PhD. Epidemiologist, Clinical Research Unit, Instituto Nacional de Saúde da Mulher, da Criança e do Adolescente Fernandes Figueira, Fiocruz, Rio de Janeiro (RJ), Brazil.

**Keywords:** Premature birth, Bronchopulmonary dysplasia, Cognition, Risk factors

## Abstract

**CONTEXT AND OBJECTIVE:**

Children born prematurely often have worse cognitive performance than those born at term regarding skills such as memory, attention and processing speed. Bronchopulmonary dysplasia may compromise cognitive development. The aims here were: a) To describe the cognitive performance of preterm infants with very low birth weight; b) To investigate its association with bronchopulmonary dysplasia adjusted for sociodemographic, neonatal and post-neonatal factors.

**DESIGN AND SETTING:**

Cross-sectional study developed in a public tertiary-care hospital.

**METHODS:**

To evaluate cognition among 112 children, we applied an intelligence scale (Wechsler scale). The average scores for children with and without bronchopulmonary dysplasia were compared across the five domains of the scale. Associations with bronchopulmonary dysplasia were investigated for domains that showed significant differences between the two groups. Associations between exposure and outcome were estimated via multivariate logistic regression.

**RESULTS:**

There were no differences in averages for the full-scale intelligence quotient, verbal intelligence quotient, performance intelligence quotient and general language composite domains. The processing speed quotient was the only domain that presented a significant difference between the two groups (P = 0.02). Among the children with bronchopulmonary dysplasia, low full-scale intelligence quotient was observed in 28.1%. In the multivariate analysis, bronchopulmonary dysplasia (odds ratio: 3.1; 95% confidence interval: 1.1-8.7) remained associated with the outcome of processing speed quotient.

**CONCLUSION:**

Bronchopulmonary dysplasia was an independent risk factor for alteration of the processing speed quotient.

## RESUMO

**CONTEXTO E OBJETIVO:**

Crianças nascidas prematuras com frequência apresentam pior desempenho cognitivo que as nascidas a termo em habilidades como memória, atenção, velocidade de processamento. A displasia broncopulmonar pode comprometer o desenvolvimento cognitivo. Os objetivos aqui foram: a) Descrever o desempenho cognitivo de crianças nascidas prematuras com muito baixo peso; b) Investigar sua associação com a displasia broncopulmonar ajustada para fatores sócio-demográficos, neonatais e pós-neonatais.

**TIPO DE ESTUDO E LOCAL:**

Estudo transversal desenvolvido em hospital público de cuidados terciários.

**MÉTODOS:**

Para a avaliação cognitiva de 112 crianças, aplicamos a escala de inteligência (Wechsler scale). Foram comparadas as médias dos escores das crianças com e sem displasia broncopulmonar nos cinco domínios da escala. A associação com a displasia broncopulmonar foi investigada para os domínios que apresentaram diferença significativa entre os dois grupos. A associação entre a exposição e o desfecho foi estimada por regressão logística multivariada.

**RESULTADOS:**

Não houve diferença entre as médias dos domínios do quociente de inteligência total, quociente de inteligência verbal, quociente de inteligência de execução e composto de linguagem geral. O quociente de velocidade de processamento foi o único domínio que apresentou diferença significativa entre os dois grupos (P = 0,02). Entre as crianças com displasia broncopulmonar, quociente de inteligência total baixo ocorreu em 28,1%. Na análise multivariada, a displasia broncopulmonar (*odds ratio*: 3,1; intervalo de confiança: 1,1-8,7) permaneceu associada ao desfecho quociente de velocidade de processamento.

**CONCLUSÃO:**

A displasia broncopulmonar foi um fator de risco independente para alteração no quociente de velocidade de processamento.

## INTRODUCTION

Premature infants may suffer from pulmonary and brain injury, along with infections early in life, thus making them more vulnerable to neurodevelopmental abnormalities.^1^ In comparison with full-term children, premature children present worse cognitive performance^1^^,^^2^^,^^3^ in skills such as memory, attention, processing speed and representational competence.^4^

In developing children, inefficiency in acquisition of elementary skills, such as attention and processing speed, can have a significant influence on their development of other more complex skills. These elementary skills form the basis from which other cognitive abilities develop. Academic performance and cognitive skills within children’s future lives are influenced by executive functions as well as processing speed. It is important to identify early problems within each of these elementary skills, so as to intervene and potentially limit more generalized cognitive deficits.^5^

In Brazil, follow-up studies on children born prematurely have been conducted.^6^^,^^7^^,^^8^^,^^9^^,^^10^^,^^11^ However, little information on outcomes from children with neonatal morbidity is available, including on its repercussions on cognitive development and its associations with less favorable socioeconomic and care conditions.

Bronchopulmonary dysplasia is a chronic respiratory disease that affects children born prematurely who undergo prolonged ventilatory assistance. This condition leads to readmissions to hospital, abnormalities of motor and cognitive development and high mortality.^12^^,^^13^^,^^14^ In a cohort of children up to eight years of age who were born with very low birth weight, those with bronchopulmonary dysplasia presented worse performance on scales evaluating intelligence.^15^

Knowledge of the areas of cognition most affected among preterm infants with bronchopulmonary dysplasia may provide parameters for early interventions that might assist them in future performance.

## OBJECTIVE

The objectives of this study were: a) to evaluate the cognitive development of preterm infants with very low birth weight; and b) to compare the results from the different domains of the Wechsler Preschool and Primary Scale of Intelligence (WPPSI-III)^16^ between patients with bronchopulmonary dysplasia and those who did not develop bronchopulmonary dysplasia during their neonatal period. We sought to determine whether bronchopulmonary dysplasia had an effect that was independent from other risk factors (sociodemographic, neonatal and post-neonatal), across the different domains of the scale.

## METHODS

### Study design and inclusion criteria

We conducted a cross-sectional study on a prospective cohort of very low birth weight preterm infants who were followed up at a public tertiary care unit. The criteria for including children in the study were that they needed to have been born prematurely with birth weight lower than 1500 g and gestational age less than 37 weeks, between 2004 and 2008. The participants were selected during the period of neonatal hospitalization, by a trained physician.

Patients with genetic syndromes, malformations and congenital infections, and children who did not follow the cognitive evaluation protocol on the WPPSI-III^16^ scale were excluded from the study.

This study was approved by the Research Ethics Committee of the Fernandes Figueira Institute (CAAE 0005.0.008.000-06). The adults responsible for the children who were evaluated provided informed consent for participation in the research.

### Main outcome evaluated

The main outcome from the study was the level of cognitive development at preschool age and the main exposure of interest was bronchopulmonary dysplasia.

After discharge from the neonatal unit, the children continued to be followed up at the Newborn Care Outpatient Clinic until reaching 11 years of age and were assessed by a pediatrician and a physiotherapist regarding their overall development. These children routinely underwent cognitive assessments over the first three years of life (Bayley scale), and at preschool age and school age on the Wechsler scale.

When they reached preschool age, they underwent a cognitive evaluation by a psychologist, who applied the WPPSI-III^16^ while unaware of the children’s clinical history. Thus, the evaluator was blinded regarding this exposure condition. The WPPSI-III evaluates children aged between 4 years and 7 years and 3 months. It was applied in an appropriate room, in the presence of the person in charge of the child. To undergo the test, these children needed to be in good health, without any complaints, and needed to be collaborative. The duration of the test application was approximately 60 minutes.

The WPPSI-III provides standardized scores corrected according to age. The scale evaluates cognitive functioning in five domains, of which four are specific domains: verbal intelligence quotient (IQ), performance IQ, processing speed quotient and general language composite. Together, these make up the fifth domain: the full-scale IQ. This total score is described qualitatively according to the level of performance and is classified as follows: extremely low: score below 70; borderline: between 70 and 79, average-low: between 80 and 89; average: between 90 and 109; average-high: between 110 and 119; high: between 120 and 129; and very high: 130 or more.^16^

In our study, the score was divided into two categories. Scores on the full-scale, verbal, execution, processing speed and general language composite scales of less than 80 were considered to represent “low IQ”.^17^ Scores in these specific domains that were greater than or equal to 80 were considered to represent “medium/high IQ”. Regarding subtests, scores below 7 were considered “low” and those greater than or equal to 7 were considered “adequate”. This scale has not yet been validated for the Brazilian population, although it is widely used.

### Other variables evaluated

The date of the last menstrual period or an obstetric ultrasound scan performed during the first trimester was used to estimate gestational age. In the absence of this information, the method described by Ballard et al.^18^ was then applied by a pediatrician on the first day of life of the baby. Weight adequacy for gestational age was based on Alexander et al.^19^

The neonatal variables included: type of birth, sex, birth weight, adequacy of weight for gestational age, gestational age, ventilatory assistance, duration of ventilatory assistance, duration of oxygen therapy and clinical intercurrences during hospitalization (respiratory distress, septicemia, apnea, pneumonia, cerebral hemorrhage and abnormalities on cerebral ultrasound scans).

Septicemia was considered to be present in cases of positive blood culture. The degree of cerebral hemorrhage was evaluated by means of cranial ultrasonography, in accordance with the classification of Papile et al.^20^ The result from the ultrasound scan that presented the highest degree of hemorrhage was used in our study. The cerebral ultrasonography was considered abnormal in the presence of at least one of the following lesions: cerebral hemorrhage, increased echo density in the periventricular white matter region, periventricular leukomalacia or ventricular dilatation. Bronchopulmonary dysplasia was defined as dependence on oxygen therapy for a period greater than or equal to 28 days.^21^

Maternal/family data were obtained through interviews with the mothers during the first consultation at the follow-up clinic and these included family income, maternal age and maternal schooling. We also collected post-neonatal data on breastfeeding, respiratory morbidity (repetitive wheezing or pneumonia or hospitalization) and whether the child had attended preschool.

Data collection began in 2004 and was completed in 2012.

### Statistical analysis

We estimated a required sample size of 109 children (32 with bronchopulmonary dysplasia and 77 unexposed), based on and considering the following parameters: prevalence ratio for cognitive alteration of 2.2;^22^ incidence of bronchopulmonary dysplasia of 30%; cognitive alteration of 25% in the non-exposed population;^22^ significance level of 5%; and power of 80%.

We calculated the prevalence of abnormalities of cognitive development in the age group between 4 and 7 years and 3 months. To analyze the difference between the proportions, we performed the chi-square test. The differences in average cognitive scores between children exposed and not exposed to bronchopulmonary dysplasia for the five domains of the scale were then ascertained. The statistical test for average differences was Student’s t test. The statistical significance level was taken to be 5%.

Multivariate analysis was performed to investigate the relationships within each domain of the cognitive scale (main outcomes) that presented significant differences between the groups exposed and not exposed to bronchopulmonary dysplasia.

Associations between the exposure variable (bronchopulmonary dysplasia) and the cognitive outcomes were estimated by odds ratios (OR) and 95% confidence intervals (95% CI) using unconditional multivariate logistic regression. The neonatal characteristics (sex, birth weight lower than 1,000 g, gestational age less than 28 weeks, type of delivery, cerebral hemorrhage, abnormal brain ultrasonography, septicemia and small for gestational age), post-neonatal characteristics (breastfeeding and attending preschool) and sociodemographic characteristics (maternal age, maternal schooling and family income) of the population were investigated as confounders.

Variables showing associations both with primary exposure (bronchopulmonary dysplasia) and with cognitive outcomes, with a significance level lower than 0.20, were considered to be potential confounding factors. Using logistic regression, we investigated variations in the magnitude of the association between bronchopulmonary dysplasia and the main outcome when adjusted for each of these study variables that had been defined as potential confounding factors in the previous stage.

The variables for which adjustment caused a change to the OR of the main exposure variable (bronchopulmonary dysplasia) that was greater than 10% were selected for inclusion in the multivariate model. The variables that, after adjustment, presented associations for which the significance level was less than 0.05 were kept in the final model. The presence of interactions between covariates and the main exposure was also investigated.

## RESULTS

**Figure 1.**
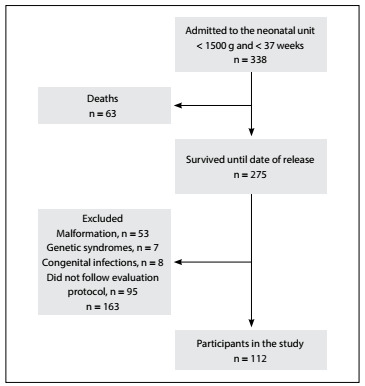
Flowchart of premature children involved in the study.

Our population comprised 112 children (Figure 1). The maternal, neonatal, post-neonatal and social characteristics of the children who complied with and did not comply with the protocol were compared, and no significant differences were found. The characteristics of the study sample and a comparison between the children who developed bronchopulmonary dysplasia and those without the disease are presented in Table 1.

**Table 1.**
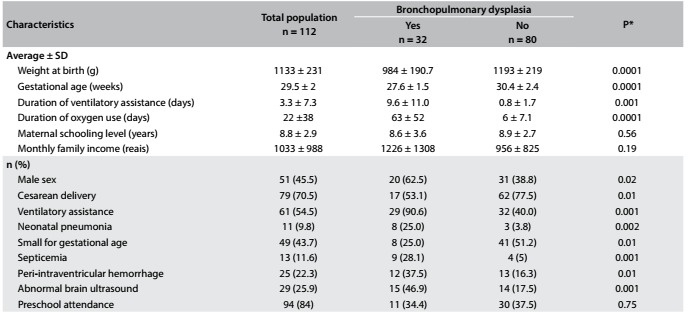
Characteristics of children born prematurely, with and without bronchopulmonary dysplasia

*comparison between characteristics of children with and without bronchopulmonary dysplasia.

Among the 32 children affected by bronchopulmonary dyspnea, oxygen treatment was administered to 15 of them at a corrected age of 36 weeks. Eight of the children with bronchopulmonary dysplasia received corticosteroids during the neonatal period.

The average maternal age was 27 years. Regarding socioeconomic characteristics, 27.6% of the mothers of these children had not completed elementary education and 8.9% of the families had incomes below the minimum wage.

The frequency of respiratory morbidity up to the age of 24 months was significantly higher among the children with bronchopulmonary dysplasia (75% versus 53%). The children were breastfed for an average period of 4.4 ± 9.1 months and those with bronchopulmonary dysplasia were breastfed for a shorter period of time (1.8 ± 4.2 months) than those without the disease (5.5 ± 10.2 months) (P = 0.02).

### Evaluation of cognitive development

At the time of application of the scale, the average age of the children evaluated was 5 years and 6 months (n = 112). The average full-scale IQ score was 86.8 (standard deviation, SD: 12.3); verbal IQ, 85.5 (SD 10.4); performance IQ, 92.2 (SD 13.8); processing speed quotient, 89.2 (SD 13.6); and general language composite, 89.4 (SD 14.2). We found that the average full-scale IQ and verbal IQ were close to one standard deviation from the average (85). In relation to subtests of the intelligence scale, all scores were below 10 and the subtest “matrix reasoning” was below 7.

Low full-scale IQ scores were obtained from 22.3% of the children and 5.3% had extremely low IQ. Low full-scale IQ scores was found in 28.1% of the children with bronchopulmonary dysplasia and in 20.0% of those without bronchopulmonary dysplasia. There was no significant difference between these proportions.

The average full-scale IQ was similar between children with or without bronchopulmonary dysplasia, but the average processing speed quotient was lower among the children affected by this than among those without bronchopulmonary dysplasia, as shown in the subtests that form this domain: “coding” and “symbol search”, in which the children with bronchopulmonary dysplasia had significantly worse performance (Table 2).

**Table 2.**
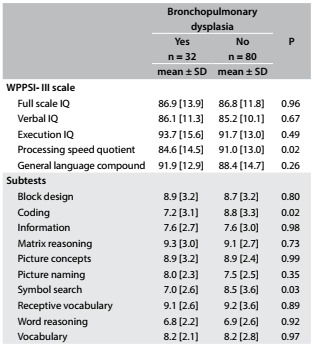
Cognitive performance scores at preschool age among children born prematurely, with and without bronchopulmonary dysplasia

SD = standard deviation; IQ = intelligence quotient.

The average processing speed quotient among the children with bronchopulmonary dysplasia who underwent corticosteroid therapy during the neonatal period (n = 8) (76.6; SD: 5.5) was significantly lower than that of the children with bronchopulmonary dysplasia who did not undergo corticosteroid therapy (n = 24) (87.4; SD: 15.6).

The average scores of the children with and without bronchopulmonary dysplasia in the five domains of the cognitive scale only showed a significant difference in the domain of processing speed quotient. Therefore, associations with bronchopulmonary dysplasia at the multivariate analysis stage were only investigated for this domain.

We evaluated whether bronchopulmonary dysplasia had an effect on the outcome of processing speed quotient independently of the other factors. After adjustment for each of the confounding variables, the following variables were selected for the multivariate model: bronchopulmonary dysplasia, male sex and septicemia.

After controlling for these risk factors, male sex presented OR 2.84 (CI: 1.08-7.43), sepsis presented OR 1.26 (CI: 0.30-4.90) and bronchopulmonary dysplasia (main exposure) remained significantly (P = 0.03) associated with low processing speed quotient (OR: 3.10; CI: 1.10-8.70). No presence of any interaction between these variables and the main exposure variable was identified.

## DISCUSSION

Bronchopulmonary dysplasia has been shown to be an independent risk factor for cognitive alteration of skills relating to processing speed. The mechanisms that lead children with bronchopulmonary dysplasia to developmental delay are multifactorial and include chronic hypoxia and poor environmental stimulation.^13^

Bronchopulmonary dysplasia has been identified as a risk factor for several neurocognitive domains including working memory, visual-motor coordination and attention.^23^ The clinical complications to which they are exposed, along with the socioeconomic vulnerability accompanying prematurity and low birth weight, predispose these children to changes in motor and cognitive development.^15^

There are few studies on the preschool and school cognition of preterm infants with very low weight belonging to Brazilian cohorts. Méio et al.^7^ used the WPPSI R scale (i.e. a previous version of the scale used in the present study) and found lower IQ in the total score (75), verbal score (78.6) and performance score (77) among preterm children at the same preschool institution who were born during the preceding decade, in comparison with the results from the present study and those in the worldwide literature. Over the last decade, there have been changes in neonatal care, including decreased time spent using mechanical ventilation, which may have led to better results more recently.^24^

Espírito Santo et al.^8^ found results similar to ours and reported that preschoolers born with very low birth weight had full-scale IQ, verbal and performance scores that were classified as medium-low.

Better performance was observed in the early 1990s, in a study by Anderson et al.^25^ in which the Wechsler scale was applied to a cohort of extremely preterm infants who were tested at eight years of age, with an average IQ of 93. In this population of premature infants, 52% of the mothers had less than 12 years of schooling and 48% were reported as having low socioeconomic status. Gnigler et al.^23^ evaluated a population of very low birth weight preterm children at the age of five years, who were born between 2003 and 2006, and obtained an average total IQ of 98.4 by means of the WPPSI- III scale.

In the present study, the average total IQ obtained through the WPPSI-III scale was 86.8 (SD: 12.3), i.e. lower than that found in the studies cited above. Moreover, almost a quarter of the total population and a third of those with bronchopulmonary dysplasia presented low IQ.

We found a high percentage of low schooling levels among the mothers of our study, such that 92% of them had attended school for less than 12 years, which may partially explain our lower cognitive outcomes. The results from more recent studies have suggested that socioeconomic factors would be more strongly associated with preschool cognition than biological factors,^2^^,^^26^ and that maternal schooling is one of the most prominent factors.^10^ Although there was no difference in maternal schooling between our study groups (with and without bronchopulmonary dysplasia), the average of eight years of schooling was lower than the level reported worldwide.^25^ Around 28% of the mothers in our study had not completed elementary education, which may at least partially explain the poor environmental stimulation to which these children were exposed. It is believed that greater schooling can improve professional qualifications and employment opportunities and, consequently, increase family income. In addition, higher levels of knowledge would influence child-related care practices.^27^

Performance in the “symbol search” and “coding” subtests that were used to measure the processing speed quotient was lower among the children with bronchopulmonary dysplasia. This reflected their impairments of attention, short-term memory, concentration, planning ability, motor visual coordination, motivation and learning. Higher frequencies of attention problems and learning impairment among preterm infants with bronchopulmonary dysplasia than among those without bronchopulmonary dysplasia were also shown in another study.^28^

It is noteworthy that some of the children with bronchopulmonary dysplasia in the present study had received corticosteroid therapy during the neonatal period. It has been reported that use of dexamethasone may alter the synaptic plasticity of the hippocampus and the formation of associative memory.^29^ Gnigler et al.^23^ found that children with bronchopulmonary dysplasia who received corticosteroid treatment were at higher risk of developing a reduction in processing speed. It is also worth mentioning that children with a history of bronchopulmonary dysplasia frequently have respiratory problems in the first years of life.^30^^,^^31^ These may exacerbate their episodes of hypoxia, thereby causing repercussions relating to their future cognition and processing speed quotient. This confirms our results, since 75% of the children with bronchopulmonary dysplasia presented respiratory morbidity for up to 24 months, compared with 53% of children without the disease. In a study on the effects of perinatal risk factors on the brain structure of preterm infants, Thompson et al.^32^ found that bronchopulmonary dysplasia was associated with an overall reduction of volume in the grey and white matter regions. Reductions in white matter volume may be responsible for diminishing processing speed.

In a study that made comparisons with full-term infants, preterm infants had worse outcomes in skills such as memory, processing speed, representational competence and attention, as well as lower IQ.^4^ It was suggested that this difference might be represented by a model involving a cascade of effects: prematurity → elementary cognitive processes (processing speed and attention) → complex processes (memory and representational competence) → intelligence quotient.

In order to study the impact of specific neuropsychological measures on academic achievement among children who were born preterm, Mulder et al.^33^ studied these children in relation to full-term controls at the ages of 9 to 10 years, with assessments that measured processing speed, executive function and IQ. They concluded that processing speed and working memory were significant predictors of overall academic achievement, such that these were important underlying factors for academic achievement among very low-weight children. They reported that all the significant differences between the groups, in terms of academic level, could be explained by processing speed. They suggested that specific rapid processing speed tests could be used as effective screening tools for assessing which children are at risk of potentially presenting educational problems and thus should be referred for complete neuropsychological evaluation. Therefore, identification of children with reduced processing speed is important in order to be able to provide special care and support, as well as to prevent further problems in school performance.^23^

Although the fact that our study population came from a single hospital may be a limitation to our study, our results are consistent with populations with similar characteristics, as described above. A larger sample size might have contributed towards a greater number of factors in the multivariate model, but the sample that was available had enough power to evaluate the association with bronchopulmonary dysplasia.

Another limitation of our study is that the Wechsler scale (WPPSI-III) has not yet been validated for the Brazilian population. Therefore, the results should be interpreted with caution, since cross-cultural adaptation issues may influence the attributes that are considered for classifying normal performance.

The present study makes a contribution towards identifying the cognitive domains that present differences between children with and without bronchopulmonary dysplasia in populations of preterm infants with very low birth weight. The difference in processing speed quotient that was obtained suggests that bronchopulmonary dysplasia may compromise this cognitive ability.

Long-term follow-up on these children, including application of neurocognitive tests, is important for identifying the children who are at risk of unsatisfactory academic performance. Moreover, knowledge of cognitive limitations provides parameters for family guidance and for pedagogical/educational interventions that may assist these children in their future lives.

## CONCLUSION

This study population, from socially vulnerable families with high biological risk, presented full-scale intelligence quotient and verbal intelligence quotient within the range classified as low-average. Bronchopulmonary dysplasia was shown to be an independent risk factor for cognitive performance, with a lower score in the dimension of the processing speed quotient.
